# Amelanotic Malignant Melanoma: A Case Report

**DOI:** 10.7759/cureus.41665

**Published:** 2023-07-10

**Authors:** Kunal Karmilkar, Richard F Norem

**Affiliations:** 1 Anatomy, Edward Via College of Osteopathic Medicine, Louisiana Campus, Monroe, USA; 2 General Surgery, Rapides Regional Medical Center, Louisiana, USA

**Keywords:** full thickness skin graft, mass resection, amelanotic malignant melanoma, malignant melanoma metastasis, amelanotic melanoma

## Abstract

Amelanotic malignant melanoma (AMM) is a skin cancer that arises from mutated melanocytes that lack pigmentation. AMM represents 2-8% of all malignant melanomas. This rare subtype is difficult to diagnose clinically as it mimics other benign skin lesions. AMM can occur in any part of the body with various presentations and has a predilection for male gender and fair skin tones. We present a case report of a 62-year-old Caucasian male with AMM of the right lower extremity. The patient presented with a painless nodule on his right lower extremity that rapidly increased in size for seven months with no signs of malignancy, such as fever, night sweats, fatigue, bruising, weight loss, or headache. Simultaneously, the patient presented with right inguinal lymphadenopathy and pitting edema of the right lower extremity. The patient had a previous medical history of basal and squamous cell carcinoma and psoriasis with no personal or family history of melanoma. The mass was excised and sent to a pathologist along with a right inguinal sentinel lymph node biopsy. The final pathology report revealed an ulcerated AMM on the right lower extremity and a positive node for melanoma with a metastatic deposit. The patient underwent adjuvant immunotherapy resulting in the clearance of the cancer cells. This report highlights the importance of early diagnosis, appropriate surgical management, and adjuvant therapy to improve the prognosis of this rare melanoma subtype.

## Introduction

Malignant melanoma (MM) is a deadly type of skin cancer that arises from melanocytes, the pigment-producing cells of the skin. Amelanotic malignant melanoma (AMM) is a subtype of melanoma that lacks pigmentation, making it difficult to diagnose clinically. AMM accounts for 2-8% of all MMs and has a poor prognosis due to its advanced stage at presentation and delayed diagnosis [1,2]. Here, we present a rare case of AMM of the right lower extremity in a 62-year-old Caucasian male, highlighting how early diagnosis, appropriate surgical management, and adjuvant therapy are crucial in improving its prognosis.

## Case presentation

A 62-year-old male presented to the general surgery clinic as a new referral. The patient had developed a painless nodule on the superolateral aspect of his right lower extremity. The lesion had been present for seven months prior to his first visit with a dermatologist. The lesion was a nonhealing ulcer rapidly increasing in size which justified his search for medical care. The patient also complained of lymphadenopathy in his right groin. He had a history of multiple benign skin cancers, which were either basal or squamous in origin, not melanotic. These prior lesions were excised and nonrecurring. There was no family history of skin cancer, specifically, melanoma. The patient also suffered from psoriasis for which he only uses topical lotions and creams as needed. Tobacco use ceased over 30 years ago. He did not take any medication. The patient reported normal appetite, steady weight, no pain, fever, chills, or sweats. No gastrointestinal changes including no blood in the stool or urine.

**Figure 1:**
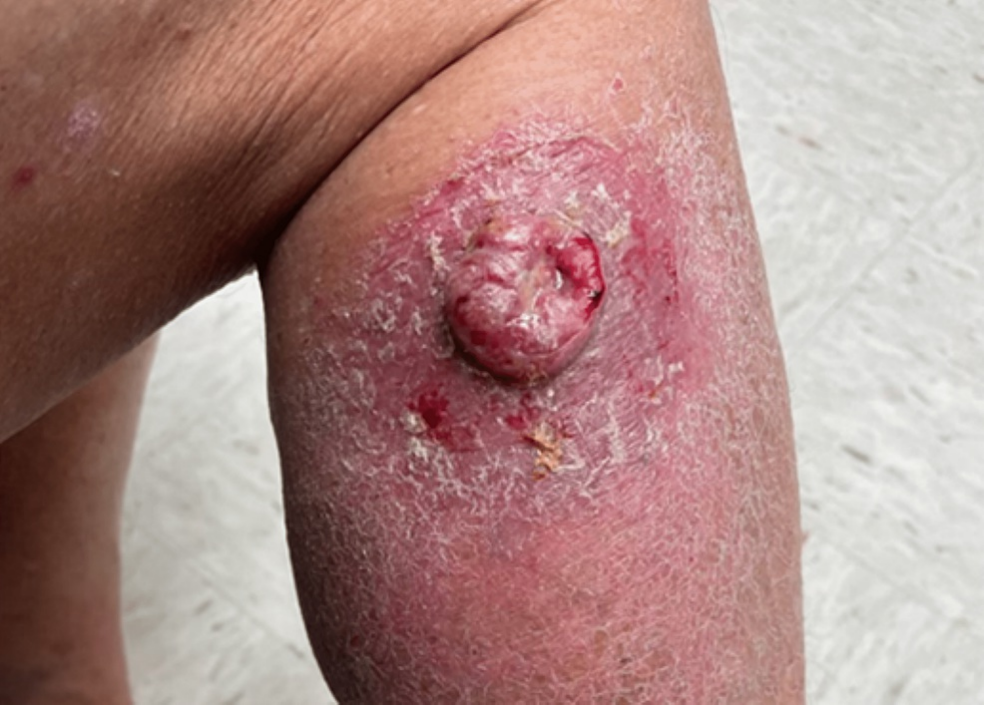
Preoperative specimen of amelanotic malignant melanoma on the right lower extremity.

Physical exam was unremarkable except for a large 5 x 5 cm lesion on the right lower extremity, a palpable large lymph node in the right groin, and 3+ pitting edema in the right lower extremity. The lesion appeared as a pink solitary hypergranulation-type nodule with irregular borders with a scaly erythematous background (Figure 1). A shave biopsy of the lesion was taken and three days later it returned as a neoplasm in the dermis composed of aggregates of atypical squamous cells, many of which were attached to the overlying epidermis, which also contained atypical keratinocytes. Focal cornification in the aggregates of neoplastic cells, and the atypical cells extend into the lower half of the reticular dermis with an ulcerated surface (Clark Level IV). Immunoperoxidase stains for S-100 protein and SOX10 were markedly positive throughout this neoplasm consistent with AMM. Stains for CD10, p40, and p63 were negative and a Breslow depth of 1.5 mm was identified.

**Figure 2:**
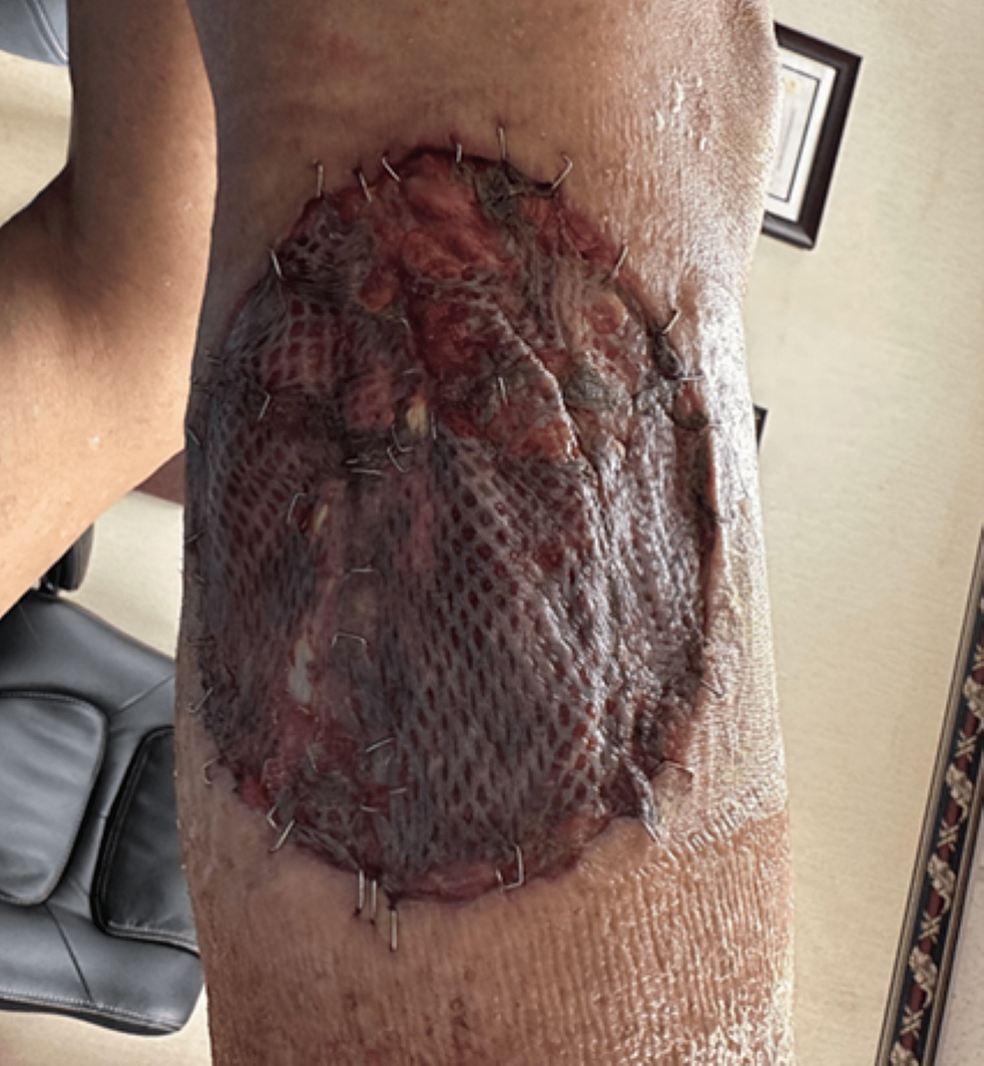
Post-operative skin graft of right lower extremity at one week follow-up.

The patient then underwent a scheduled excision of the right lower extremity AMM with 2 cm margins, as well as placing a split-thickness graft to the area. A sentinel lymph node biopsy was performed in lieu of dissection to avoid worsening of lower extremity edema. Pathology from the lesion again revealed American Joint Committee on Cancer (AJCC) stage IIIC AMM and the sentinel lymph node was positive for melanoma with the largest metastatic deposit being 2 cm. Surgical margins of 2 cm were negative with no extra nodal extension. The patient was followed clinically post-operatively for dressing changes and to assess healing progress. At one week follow up the skin graft was healing appropriately (Figure 2). Staging workup with PET scan and brain MRI were unremarkable; therefore, adjuvant nivolumab 90 mg intravenous (IV) and ipilimumab 270 mg IV were administered q3 weeks.

## Discussion

The least to most common melanoma subtypes are acral lentiginous melanoma (1-2%), lentigo maligna melanoma (5-10%), nodular melanoma (20%), and superficial spreading melanoma (70%) [3]. Nodular melanoma, which can be pigmented or amelanotic, has a rapid growth rate and a worse prognosis than more common melanoma subtypes [4]. Our patient’s AMM falls under the nodular subtype and is particularly challenging because it can be easily mistaken for other non-melanocytic lesions, leading to delayed diagnosis and treatment. 

The clinical presentation of AMM is variable and can range from a papule or nodule to an ulcerated or hemorrhagic lesion. Hence, the diagnosis of AMM is mostly based on histopathological examination and immunohistochemical staining. Histological features of AMM include epithelioid, spindled, or desmoplastic morphology with prominent nucleoli, a high mitotic rate, and an absence of pigmentation [5]. Anti-S-100 has a high sensitivity for AMM and is commonly utilized to analyze MM immunohistochemically, but other stains, such as vimentin, Melan A, and HMB-45 have also been reported [6,7]. Our patient also presented with a right inguinal sentinel lymph node, which is why the sensitive and specific marker SOX10 was used to identify metastatic MM. Willis et al. reported that SOX10 successfully identified metastatic melanoma in 58 out of 58 positive MM cases [7].

The primary treatment for localized AMM is surgical excision with a wide safety margin. The recommended margin size for AMM is controversial as it depends on the development of the lesion, thickness, and depth of invasion. However, although not evidence-based, guidelines from the Annals of Surgery recommend melanomas greater than 2 mm be excised with 2 cm margins [8]. Sentinel lymph node biopsy is recommended for thicker lesions or those with high-risk features. Cascinelli et al. described how the five-year survival of patients receiving an immediate lymph node dissection at presentation was higher than those who received a delayed lymph node dissection. This offered increased survival in those with node metastases [9].

In advanced cases, adjuvant therapy may also be considered in patients with high-risk features, such as ulceration, high mitotic rate, or lymph node involvement. Systemic therapy with immune checkpoint inhibitors and targeted therapies may be required. Nivolumab and ipilimumab have shown promising results in the treatment of AMM [10]. However, other studies have reported that postoperative radiotherapy only had beneficial effects on the ameliorating locoregional spread and no data to date suggested an overall survival benefit [11].

AMM is associated with a less favorable prognosis compared to other subtypes of melanoma, largely attributable to a heightened incidence of lymph node involvement, distant metastasis, and consequent mortality. The rapid rate of progression, advanced tumor stage at diagnosis, and delayed detection may entirely account for the increased severity of this disease [12]. Thus, early identification and prompt therapeutic intervention are pivotal in enhancing the prognosis of AMM. Moreover, the scarce amount of effective diagnostic and treatment modalities underscores the need for further research to advance the development of targeted strategies.

## Conclusions

AMM is a rare subtype of melanoma that requires a high index of suspicion to ensure a timely diagnosis. In order to improve the prognosis of AMM, early detection, precise surgical excision, sentinel lymph node biopsy, and systemic therapy are essential. Increased awareness and extensive comprehension of AMM among clinicians can lead to favorable outcomes for patients afflicted with this aggressive disease.
